# Anticipating responses to climate change and planning for resilience in California’s freshwater ecosystems

**DOI:** 10.1073/pnas.2310075121

**Published:** 2024-07-29

**Authors:** Mary E. Power, Sudeep Chandra, Peter Gleick, William E. Dietrich

**Affiliations:** ^a^Department of Integrative Biology, University of California, Berkeley, CA94720; ^b^Department of Biology, University of Nevada, Reno, NV89557; ^c^Pacific Institute, Oakland, CA946124; ^d^Earth and Planetary Science, University of California, Berkeley, CA94720

**Keywords:** freshwater ecosystems, rivers, lakes, wetlands, critical zone

## Abstract

As human-caused climate changes accelerate, California will experience hydrologic and temperature conditions different than any encountered in recorded history. How will these changes affect the state’s freshwater ecosystems? Rivers, lakes, and wetlands are managed as a water resource, but they also support a complex web of life, ranging from bacteria, fungi, and algae to macrophytes, woody plants, invertebrates, fish, amphibians, reptiles, birds, and mammals. In much of the state, native freshwater organisms already struggle to survive massive water diversions and dams, deteriorating water quality, extensive land cover modification for agriculture and urban development, and invasions of exotic species. In the face of climate change, we need to expand efforts to recover degraded ecosystems and to protect the resilience, health, and viability of existing ecosystems. For this, more process-based understanding of river, lake, and wetlands ecosystems is needed to forecast how systems will respond to future climate change and to our interventions. This will require 1) expanding our ability to model mechanistically how freshwater biota and ecosystems respond to environmental change; 2) hypothesis-driven monitoring and field studies; 3) education and training to build research, practitioner, stewardship, and policy capabilities; and 4) developing tools and policies for building resilient ecosystems. A goals-driven, hypothesis-informed collaboration among tribes, state (and federal) agencies, nongovernmental organizations, academicians, and consultants is needed to accomplish these goals and to advance the skills and knowledge of the future workforce of practitioners, regulators, and researchers who must live with the climate changes that are already upon us and will intensify.

California’s diverse aquatic ecosystems range from desert springs to mountain lakes, wet meadows and streams, rainforest streams, and large lowland rivers, reflecting the state’s 10° of latitude, its elevation range from 86 m below sea level to 4,421 m above, its geologic heterogeneity, and coastal to desert to alpine climate zones. California’s freshwaters have been extensively modified and diverted to support the state’s growing human population and agricultural economy, as well as urban and industrial development. Both natural and heavily modified freshwater ecosystems in California face intensifying “weather whiplash” from a warmer planet: more prolonged, hotter droughts interspersed with torrential atmospheric rivers and megafloods (1). Along with these come rising temperatures and sea levels, and modified snowfall and snowmelt dynamics. Planning is underway to sustain human infrastructure and enterprises, but these anticipated changes also demand more advanced understanding of how freshwater ecosystems will respond, so actions taken will effectively enhance ecosystem resilience.

In this Perspective, we discuss the range of hydroclimatic conditions under which freshwater ecosystems and organisms in California evolved, and currently observed impacts of climate change on hydrology. Then, we describe climate sensitivity in three California freshwater ecosystems: rivers of California’s North Coast Range, California’s high mountain lakes, and rivers and floodplains of the Central Valley. We chose both North Coast rivers and high mountain lakes as ecosystems that are less impaired by dams, pollution, or water extraction than lowland freshwaters or rivers of the Central Valley, the Sierra, or southern California. With fewer human impacts, ecosystem responses to climate change might be more detectable, and resilience measures might protect valuable natural ecosystems and native biota. We also review work and insights on wetlands of California’s Central Valley, as these were once the dominant aquatic habitat of the state. Recovering California wetlands would ameliorate hydrologic extremes anticipated under climate change, and could recover valued native plants, birds, fish, and wildlife populations. While basic ecosystem features and processes have been well studied in these and other freshwater ecosystems of California, we must ramp up our ability to predict states of specific freshwater ecosystems under future new scenarios. This requires that we quantify causal linkages between climate variables and processes that structure ecosystems. Watershed-scale understanding of ecosystems, with improved hydrologic models and monitoring of subsurface and snowpack storage dynamics, hydrologic flow paths, temperature variations, and relative humidity is essential. Building on the considerable current statewide efforts to track and anticipate ecosystem change, California should establish a coordinated program of modeling, monitoring and research, and training that will advance mechanistic forecasting of future aquatic ecosystem states. Investments, changes in water use and infrastructure, and new water policies that build or maintain ecosystem resilience can be designed and prioritized only if we learn how social–ecological systems will respond to managed or unmanaged change.

## California Climate as We Knew It

Most of California has a Mediterranean climate with cool, wet winters and warm, dry summers; however, five components of this strongly seasonal Mediterranean hydrograph are critical for native biota in rivers: the first fall flood pulses, wet season flow peaks, wet season baseflows, spring recession flows, and dry season baseflows (2). In the high country of the Sierra Nevada, Klamath, and Cascade mountains, winter snow precipitation builds up snowpack. Snowmelt generates high spring runoff, which diminishes through the dry summer months.

California precipitation is one of the most variable over space of any state in nation, ranging from >200 cm y^−1^ in the northwest corner of the state to <5 cm y^−1^ in its southeast corner in the Colorado Desert (3). In addition, the state’s annual variation in precipitation exceeds that of any other state. California can experience multiyear droughts followed by a year with large floods. This variation is driven by shifting offshore pressure systems that determine whether or not atmospheric rivers arrive. These are regions of concentrated water vapor streaming across the ocean that are of the same length-scale as the Mississippi, but can contain >10× more water. When atmospheric rivers arrive in California, significant winter rain falls on the Coast Range and significant snowfall is delivered to the higher mountain ranges, with the western slopes of both ranges receiving more precipitation. Atmospheric rivers can supply 30 to 50% of the state’s annual precipitation within a given year (4). But when shifts in the offshore high-pressure system deflect atmospheric rivers to the north of California, multi-year droughts can ensue, and with warming, the impact of these droughts intensifies.

## Impacts of Climate Change on Hydrologic Support for California’s Freshwater Ecosystems

The strong influence of climate change is now seen throughout California. Consistent with global trends, air temperatures have risen statewide, with some of the hottest temperatures on record in the last decade (5). Since 1950, temperatures have increase, by day and more so at night, reducing snowpack and groundwater storage by increasing relative humidity, evaporation, plant transpiration, and altering melt dynamics. While the annual average precipitation has not changed, its variability has increased along with the fraction falling as rain instead of snow in the Sierra Nevada and Southern Cascades, reducing water storage in the snowpack that supports agriculture and summer discharge into rivers, lakes, and meadows. With warming, discharge before April 1 has increased at over 630 reporting stations with long-term records in the western United States (6). Earlier snowmelt and reduced later runoff during the historical snowmelt period (April to July) has long been predicted (7) and is now evident in records from the Sacramento-San Joaquin river system in California (8). Simply stated, climate change has shifted the timing of spring snowmelt and runoff, increased winter flows and decreased spring and summer flows. These shifts have increased risks of winter flooding, challenged management and operating rules for the state’s major reservoirs, shifted river temperature regimes, and disrupted cues for the migration and spawning success of major anadromous species.

Along with ongoing changes to air temperature and the natural hydrologic pulses of water, extreme events have repeatedly struck the state, including a severe 5-y drought from 2012 to 2016, one of the wettest years on record in 2017, a follow-on 3-y drought from 2018 to 2021, and another extreme wet year in 2023. Droughts exacerbate wildfires, which affect aquatic biota and biogeochemical cycles via state changes in watershed vegetation and soils, accelerated erosion, and smoke that can cool freshwaters (9, 10). A megadrought (approximately 19% attributable to human-caused climate change, and estimated to be the driest 22-y period in over 1,200 y) depleted flows in the Colorado River (11). Changes in seasonal timing of precipitation (12) can have large consequences for the biological connections between rivers and the ocean [e.g., delaying ascent of salmon up rivers for spawning (13)]. To understand how rising temperatures, altered hydrographs, weather whiplash, and climatic extremes will affect the diverse freshwater ecosystems of California, we need to know not only their direct physiological and life history impacts on biota and ecosystem traits, but also how their indirect effects are mediated through ecological and landscape interactions.

## California’s Freshwater Ecosystems: Current State, Future Threats

Over their ~10,000-y (Holocene) histories, the 1,700 species of the state’s native freshwater biota that depend on freshwater for part or all of their life cycles (14) adapted to California’s Mediterranean hydroclimate, which developed at the end of the last global glaciation. While hydroclimatic extremes damage or destroy human infrastructure and enterprises, native freshwater biota of California survived droughts, floods, and other environmental extremes via life history, behavioral, or physiological adaptations. Now, however, California’s freshwater organisms confront rapid climate change in landscapes already drastically altered by two centuries of post-colonial land conversion. More than half of all renewable flows (runoff) in the state’s rivers have been extracted for agricultural and industrial or urban use; groundwater pumping has lowered groundwater levels (effectively mining the water), causing land subsidence, loss of river baseflows, and drying up of inland wetlands (15). Remaining lowland channels, pools, and wetlands are loaded with agrochemicals, excessive fine sediments, heat, sewage, and salinized agricultural return waters (e.g., ref. 16). Mountain lakes, streams, and wet meadows endure legacy and current impacts of logging, grazing, and fire suppression. California aquatic ecosystems are heavily invaded by exotic species, and many of these harm natives via competition, predation, or habitat modification (e.g. refs. 17–19).

Unsurprisingly, many native freshwater species are imperiled or extirpated (14) including ~80% of California’s native fishes (20). Post-colonization history in the Eel River of northwestern California is representative. Annual spawning runs of Eel River salmonids before European settlement were estimated at 800,000 fall-run chinook (*Oncorhynchus tshawytscha*), 100,000 coho (*O. kisutch*), and 150,000 winter and summer steelhead (*O. mykiss*), with abundant coastal cutthroat trout (*O. clarki*), Pacific lamprey (*L. tridentata*), and green and white sturgeon (*Acipenser medirostris, A. transmontanus*); only a few percent of these populations remain today (21, 22). The loss of native freshwater species and their natural habitats leaves human Californians—tribal members and settlers—impoverished, spiritually, culturally, physically (23), and economically. For example, if 1) Eel River returns were at historical levels (ca. 1 M fish per year), 2) fish captured averaged 20 pounds, and 3) local catches fetched $.50 per pound, a restored fishery could bring $10 M per year into a severely economically depressed region.

## Anticipating Ecosystem Response to Climate Change

Recognizing the value of freshwater biota and ecosystems, California has launched initiatives and programs centered on water, ecosystems, and mitigating or adapting to effects of land use and climate change. Efforts are underway to manage groundwater [Sustainable Groundwater Management Act (SGMA)], (24), build “water resilience” (25), plan nature-friendly management for flood control (26), develop strategies and actions to adapt to climate change (27), and conserve 30% of State lands and coastal waters in a “natural” state by 2030 (28). The California 30 × 30 program engages over 70 different federal, state, tribal, academic, NGO, and local programs. State agencies are developing tools and guidelines (e.g., refs. 29 and 30), and statewide hydrologic models (e.g., ref. 31). In addition, continued efforts to examine environmental flows and evaluate their adequacy for aquatic and riparian species under climate change are being examined with conceptual models and field studies (e.g., ref. 32). Recently, as of this writing, the State has supported a new program, COEQWAL (Equitable stewardship of California’s water in a changing climate) to create tools, data, public education, and partnerships that will inform more open and transformative discussions about water operations and management in the Sacramento-San Joaquin River system in a changing climate. Collectively, these programs and initiatives are generating valuable information about the state of California’s waters and ecosystems.

Essential to these efforts are projections of California’s future water supplies under various climate scenarios via coupled climate and hydrologic models. These models have been built on decades of research on Earth surface and atmospheric processes and feedback, extensive monitoring, and model development and testing. Community climate models now underlie our understanding and anticipation of climate change (33). Downscaling these quantitative, process-driven climate models helps us to forecast impacts at the local, large watershed scales needed for forecasting ecosystem responses (34). We now need similar collective momentum to make freshwater ecosystem science more quantitative and predictive. Ecological theory has outpaced our empirical knowledge and understanding of how and why ecological interactions in specific ecosystems have changed and how they are likely to respond to future environments (35). To prepare and adapt to climate change, California needs quantitative models framed for specific ecosystems with sufficient mechanistic insight to forecast the promise and risks of alternative restoration or resilience measures. Current ecosystem models largely rely on conceptual frameworks and statistical correlational analyses, often assuming stationarity and invariant ecological relationships. Yet, as widely observed (35), including in field studies reviewed below, ecological interactions change in strength and sometimes direction across space and time. Without mechanistic understanding of ecological context-dependence, we have little ability to predict the state of freshwater ecosystems under future climate scenarios. Important questions need to be answered: How do species performances and interactions change with changes in hydroclimatic, biotic, or abiotic conditions? How do interactions among species and environmental factors affect energy flow and nutrient cycling? What changes could tip ecosystems from one state into a very different alternative?

## Challenges of Forecasting Ecosystem Responses to Climate Environmental Change

One unresolved issue in forecasting ecological effects of climate change is the level of biological organization needed. To what degree are ecological patterns and dynamics through space and time governed by the ecophysiology of individual species, versus higher-level community or ecosystem interactions (36, 37)? Models of ecological impacts of climate change have often relied on “physiological envelopes” using current niches of individual species of plants and animals to predict how their biogeographic ranges will shift as environmental conditions are redistributed over the Earth’s surface by climate change. For example, a fish species will not survive if water warms above a certain temperature. But well before that threshold, the fish may succumb if warming increases virulence or prevalence of its parasites or pathogens, or reduces its resistance to them. Unraveling how environmental hazards, stresses, and opportunities affect performances, abundances, interactions and impacts of species requires intensive, place-based, long-term field studies over scales large enough to reveal crucial players, processes, linkages, and feedback (38). Our insights into these linkages lag behind our need for them.

Below, we discuss several California aquatic ecosystems in which multi-decadal research generated insights but also surprises, showing the need for monitoring and research continuity to test or revise current understanding as climate changes accelerate.

### Floods, Droughts, and Alternate Summer Food Webs in Rivers of California’s North Coast.

Attached algae of rivers and lakes are the primary producers fueling food webs in clear, sunlit freshwaters (39), including rivers cutting through the California Coast Range. In more hydrologically stable freshwater ecosystems like lakes, thin veneers of fast-growing, nutritious algae at the base of the food web can fuel invertebrate production that in turn feeds fish and other longer-lived, larger predators. In clear-water lakes, fast turnover of small algal producers and slower turnover of larger, long-lived predators can produce “inverted trophic (food) pyramids,” in which a large biomass of predators is supported by a miniscule biomass of algae—therefore maintaining clean water while sustaining wildlife and fish populations. But large algal proliferations do not always indicate unhealthy eutrophication. Early summer proliferation of attached algae is a normal feature of Mediterranean rivers, due to their winter flood-summer drought seasonality. A case in point is the Eel River of northwestern California. The Eel and its tributaries experience scouring winter floods that obliterate and export attached algae, along with other rock-bound biota. After bed-scouring winter floods, however, summer proliferations (largely of the filamentous green macroalga *Cladophora glomerata*) are longer (often >10 m) and more vibrant than after scour-free winters when these green algal turfs generally remain short (<0.5 m).

Attached algae proliferate in spring if winter flood scour has killed or exported large, predator-resistant aquatic insect grazers, which, like algae, suffer huge mortality and export during flood scour, but are much slower than algae to recover (40–42). As receding flows clear and warm, and days lengthen, regenerating algae experience good growth conditions and no impactful grazing. Densities of faster-growing grazing insects (e.g., midges, mayflies) build up over the summer baseflow via re-colonization, reproduction, and river contraction. These early summer taxa are mostly soft and mobile—vulnerable prey for juvenile salmonids and other predators. During post-scour summers, “top–down” predation on edible grazers indirectly affects algal biomass (40–42). In contrast, when no bed-scouring winter floods occur, large, armored, caddisflies (*Dicosmoecus gilvipes*) survive and abound the next summer (41). Invulnerable to most predators in the upper South Fork Eel, these large grazers sequester algal production and suppress algal biomass. Hence, whether at least one scouring winter flood pulse occurs or not explains much of the year-to-year variation in algal abundance, in predator effects in food chains (40, 41), and possibly in juvenile salmonid growth (43).

Algal proliferations are generally viewed as nuisances in freshwater ecosystems, but attached algal turfs and the microbes they support can be a nutritional bonanza for both river (44) and estuarine (45) consumers, fueling food chains that support salmonids. By mid-summer, *Cladophora* turfs become smothered under epiphytes: small organisms that attach and grow on their surfaces. By midsummer, *Cladophora* epiphytes are dominated by diatoms in the genus *Epithemia* that contain nitrogen-fixing endosymbionts (46). Nitrogen-fixing *Epithemia* are extremely nutritious—rich in amino acids, carotenoids, and lipids including poly-unsaturated fatty acids. *Epithemia*-smothered turfs of *Cladophora* are voraciously grazed by insect larvae (44), tadpoles (47) and when exported to the Eel estuary, small crustacea (45), all of which are consumed by fish. This salmon-supporting ecosystem, however, depends on whether summer base flows remain high enough to keep mainstem pools cool and gently flushed (48). If summer baseflows decrease to the point at which large portions of mainstem pools warm and stagnate, mats of potentially neurotoxic cyanobacteria (49) smother and consume nutrients released by the senescing diatoms and green macroalgal hosts that they overgrow. Trapped oxygen bubbles eventually pull slimy cyanobacterial mats off the substrate, and they drift down to collect along river margins and in backwater pools, where dogs encounter them. If dogs lick their fur after wading through neurotoxic cyanobacteria, they can die in convulsions within 20 to 30 min. Neurotoxic cyanobacteria in the Eel and other coastal rivers have poisoned dozens of dogs over recent years (48, 50 and references therein).

These studies link river ecosystem states to winter and summer flows. High winter flows mobilize gravel beds and increase salmon rearing success, whereas low hot summer flows stress salmon and trout and nutritious algae, and trigger blooms of harmful heat-tolerant cyanobacteria (48–50).

### Frogs, Parasites, and Heat Waves.

Temperature extremes under climate warming affect other taxa in aquatic ecosystems directly and indirectly. Egg mass surveys from 1992 to 2023 show that the South Fork Eel River within the University of California Angelo Coast Range Reserve support some of the state’s most stable populations of foothills yellow legged frogs (*Rana boylii*), California’s only river-breeding frog and a species of special concern (51). From April to mid-May, these frogs lay eggs along river margins. During this time window, egg masses or small tadpoles can be scoured away by flow pulses that sometimes occur with late season rain. Eggs and small tadpoles can also be stranded and desiccated if river stage drops too rapidly. Flow is not all that matters, unfortunately. One year, an ideal spring with gentle rains that prevented desiccation led to record numbers of hatching egg masses, but was followed by an intense summer heat wave. Tadpoles became infested with exotic thermophilic ectoparasitic crustaceans (*Lernaea cyprinacea*) which attack limb buds on metamorphosing tadpoles. Limb abnormalities (e.g., three hind legs) were seen on transformed frogs for the first time in the South Fork Eel, likely caused by the heat-enhanced parasitic infection (52). Other heat-related diseases include black spot infection of juvenile trout that increase dramatically as water warms (53).

To evaluate impacts of thermal and radiation regimes on *yellow legged frog* tadpoles, Catenazzi and Kupferberg (54) reared egg masses in identical flow-through enclosures in four streams that differed in forest canopy cover, and hence insolation and summer temperature regimes. We report the details of this experiment in *SI Appendix*, because it illustrates how experimental comparisons of ecological interactions over habitats with different ranges of conditions (temperature, flow) can lead to “predictive mapping”—inferences about how changes in future conditions will affect these interactions and their feedback to populations and ecosystems (*SI Appendix*, *Catenazzi and Kupferberg: Towards Predictive Mapping*).

### Flow, Frogs, and Recreational Rafting in River Reaches Downstream of Sierra Nevada Dams.

Knowledge of *R. boylii* life histories from long-term field censuses was crucial in interpreting their population crash in the Feather River of Lassen National Forest of Plumas Co, which drains the Sierra Nevada mountains in Northern California. A 7.6-km reach below the Cresta Dam on the Feather River was subjected to multiple summer releases for whitewater boating from 2002 to 2005, whereas further downstream, a comparable reach 8.3 km below the Poe Dam on the same river was not. Kupferberg warned in Federal Energy Regulatory Commission testimony that Cresta dam releases would devastate local *R. boylii* populations, but rafting industry representatives pointed out that after the first 2 y of releases, adult *R. boylii* frogs were still present. Knowing from her long-term population data that female reproduction peaked at age 3, Kupferberg warned that one more year of summer releases would eliminate the local frog population, and they did. [Frog populations downstream from the “control” Poe dam on the Feather River actually increased during this time (51)].

Such studies show how life histories with “storage effects” [e.g., long-lived adults who reproduce more than once, or long-lived seed banks in plants (54, 55)] confer resilience, enabling populations to recover from transient or short-term stresses. However, these long-term studies also show that storage effects can also conceal harmful impacts of management practices or consequences of climate change until such population “storage reservoirs” are used up.

### Changing Snowmelt Regimes in California’s High Mountain Lakes.

Mountain lakes, streams, rivers, and wet meadows store and supply water for small communities as well as major cities (e.g., Los Angeles, San Francisco) and agriculture in California. These mountain freshwater ecosystems also support valued native amphibians, inland trout, and birds, and buttress important regional mountain economies through recreation-based tourism (e.g., Lake Tahoe). High elevation mountain ecosystems watered by snowpack are sensitive to elevation-dependent warming (56). Under climate change, more and larger atmospheric rivers deliver well above average precipitation in short bursts, curtailing snow accumulation in lake watersheds. In addition, California’s mountain freshwater ecosystems are sensitive to both dry snow droughts (below-normal cold-season precipitation) and warm snow droughts (when little snow accumulates despite near-normal precipitation because precipitation is delivered as rain rather than snow) (57). Here, we examine causal pathways mediating climate and introduced species effects on ecosystems of Lake Tahoe and smaller mountain lakes to illustrate the interactions of climate and fundamental ecological processes affecting these lake ecosystems.

### California’s Deepest Lake: Tahoe.

Known for its cobalt blue waters and remarkable clarity, Lake Tahoe supports a tourist-based, regional economy of $5.1 billion per year (58). Clarity of Tahoe has declined by 10 m since 1967, a decline attributed to fine (<5 µm) suspended inorganic sediments washed into the lake during winter-spring runoff and to drifting open-water algae called phytoplankton (59).

In contrast to the Eel River food web, ecosystem studies in Lake Tahoe have found that grazers do not control algae (phytoplankton) due to their very low abundances in this extremely nutrient-poor (ultra-oligotrophic) lake. In experimental manipulations of nutrients and a widespread zooplankton grazer (*Daphnia*) in Tahoe and two other lakes with higher nutrient concentrations, Elser and Goldman (60) found a hump-shaped relationship between nutrient-limited ecosystem productivity and strength of grazer control by the water flea (*Daphnia* spp). Nutrients, not zooplankton at their low ambient densities, controlled phytoplankton in Lake Tahoe. Grazer control was also weak in eutrophic (extremely nutrient-rich) Clear Lake, where less edible cyanobacteria dominate phytoplankton. Only in a lake of intermediate (mesotrophic) nutrient status, Castle Lake, did grazers control phytoplankton (60).

Understanding whether algal densities are controlled by their own resources (“bottom–up limitation”) or by their consumers (“top–down limitation”) is crucial for allocating management resources and preserving lake clarity. In 2023, a press release that was widely republished in news media attributed a slight improvement in Tahoe clarity to reductions of *Mysis*, an exotic shrimp that preys on zooplankton, introduced to Lake Tahoe in 1963 to 1964 (61). Decades of evidence, including a recent experiment in Lake Tahoe (62), consistently indicates that exotic *Mysis* do not influence the concentration of particles in the water and thus clarity. Now as in the past, nutrients and fine sediments from the watershed, not zooplankton, control phytoplankton and clarity in ultra-oligotrophic Lake Tahoe. If efforts to suppress *Mysis* came at the cost of reducing efforts to decrease nutrient and fine sediment loading to the lake, Tahoe might become enriched enough for consumer control to take over algal population limitation. But by then, Tahoe’s cobalt blue waters would be long gone.

A process-driven lake clarity model (63) has guided policy makers who implemented a successful Tahoe Maximum Daily Load Program to reduce nutrients and fine (<5 µm) inorganic particles delivered from watershed runoff to the lake. As Lake Tahoe warms and watershed inputs shift, it will be critical to expand the processed-based understanding of such models to forecast current and future climate effects, both to manage the lake’s clarity and to protect its native biodiversity (64). Under the new hydroclimatic regimes, peak runoff events that deliver fine sediments from the watershed clarity have shifted from June to April, and discharge peaks are predicted to occur as early as January by the end of century (64). In addition, lake warming may affect phytoplankton in ways that threaten lake clarity. Lake warming favors a small-celled diatom, *Cyclotella* (65). Diatoms have heavy silica cell walls, so can sink out of the light zone faster as lakes warm, but *Cyclotella* is small enough to avoid this (66). Small cells with high surface:volume ratios also take up sparse dissolved nutrients more efficiently. So far, increases in *Cyclotella* have had minimal influence on lake clarity (65), but the increased phytoplankton production and its deposition on the lake bed are limiting light there for deep benthic algae and plants, reducing energy flow to benthic invertebrates and fishes.

Lake Tahoe’s clarity is tracked in the open areas away from the shore, but the lake’s nearshore edge and changes to the lake bottom are also of recent concern. Worldwide, lakes once considered pristine are greening—attached green macroalgae are proliferating on their shorelines and beds (67). The public perceives that algal growth along nearshore lake beds and beaches has increased, although this trend in Tahoe is not obvious in long-term data, which show high year-to-year variation in benthic algal biomass (68). Climate-driven changes in snowpack dynamics could affect timing of nutrient-rich groundwater influxes—more nutrient delivery when days are longer, for example, could stimulate algal growth (69). In addition, warming nearshore water temperatures are allowing invasive species like warm water fishes from the Mississippi basin to establish and expand around the lake (70). Such invaders could promote algal growth and accrual via nutrient cycling or predation on algal consumers (71). An invasive cold-water crayfish is also reducing benthic macrophyte biomass in Tahoe. These macrophytes host nutritious *Epithemia* diatoms, and serve as both food and habitat for Tahoe’s endemic invertebrates, including one of the world’s wingless stoneflies and two species of blind amphipods (72–74). Consequently, these endemic invertebrates are threatened by the invasive crayfish and perhaps the New Zealand Mud Snail, another invasive discovered in September 2023. In the last 15 y, governments and agencies have attempted to reduce densities of invasives and protect Lake Tahoe from additional exotic species introductions (71). It is not clear whether these efforts will be effective under a new climate regime. Process-based modeling based on improved understanding of controlling variables and biotic responses is needed to forecast nearshore lake responses to warming, altered timing of stream inflows, and other climate-related changes (e.g., wildfire ash deposition) that affect both nearshore and offshore biota in Lake Tahoe.

### Small Mountain Lakes.

Small mountain lakes and ponds are even more sensitive to changes to their landscapes, airsheds, and climate than large lakes. Despite warming air temperatures over the last three decades, temperatures in the shallow, wind-mixed waters of small lakes remain primarily driven by snowpack dynamics (75). The snowpack regulates the duration of winter ice cover and volume of spring flows into lakes. Observing that timing of ice-break-up was determined by winter snowpack and air temperature across 15 mountain lakes in the Sierra Nevada and Klamath Mountains, Smits et al. (76) proposed that as snowpack declines in the future, ice will break up earlier. In western mountain lakes, ice cover is predicted to break up 25 to 61 d earlier by the end of the century (77). With earlier break up, lake oxygen will increase, with variation driven by different lake morphology and basin elevations and aspects. Our ability to forecast key physical and chemical responses to hydroclimatic change is more advanced than our ability to connect such changes to ecology. Scientists have, however, unraveled how winter-spring snow and ice regimes are connected to ocean dynamics (e.g. El Nino/La Nina), followed by changing summer heat content in shallow, wind-mixed lakes—changes that will influence lake primary production (78).

Primary production (mainly photosynthetic carbon fixation) in lake food webs is driven by algae: attached algae where adequate light reaches the lake bottom (79), and drifting phytoplankton in open deeper water. The relative amount of production from attached versus planktonic algae depends on lake clarity, which in turn is influenced by nutrient concentration (80). In montane Castle Lake in California’s Siskiyou mountains, hydroclimatic extremes reduce both (81). During dry (early ice-out date and low snowpack) and wet (late ice-out date and high snowpack) years, both nearshore benthic habitats and open-water habitats have lower summertime production than during years with average snowpack and ice-out timing (81). Early ice-break-up may increase production more on lake substrates than in open water. If lake fish tracked shifting food availability, then following early ice breakup, they should feed in nearshore waters where zoobenthic prey are more abundant than pelagic zooplankton. However, prey tracking by native cold-water trout may be thwarted if early ice breakup also warms nearshore shallows to stressful temperatures (81). Forecasts of warming impacts on native fish depend on knowing both the direct effects on their physiology, and indirect effects mediated through species interactions.

Learning how longer-term stresses like droughts and shorter-term disturbances like atmospheric rivers impact watershed-to-lake connections will require more continuous monitoring of snowpack and ice phenology in basins of California’s small mountain lakes. Our current understanding is now largely based on short-term studies and a few largely unfunded, long-term monitoring programs from Northern California and the southern Sierra Nevada in Sequoia/ Kings Canyon. Monitoring of ecological, not just physical, responses is necessary to build process-based models that can guide management for resilience to future climate regimes.

### Central Valley Floodplain-River Systems.

The two great rivers draining from the Sierra Nevada and Cascade mountains to the California Delta—the Sacramento from the north, and the San Joaquin from the South—deposited their sediments across the floor of the Central Valley, creating vast freshwater wetlands. Before Europeans arrived, Native Californians foraged on abundant fish and waterfowl from rafts created from Tule reeds (*Schoenoplectus acutus*). British fur trappers introduced malaria, triggering an epidemic that killed as many as 75% of the human residents by 1846 (82). With the onset of the Gold Rush, settlers drained wetland “wastelands” and converted much of the land around the Sacramento Valley, the Sacramento-San Joaquin Delta, and the Tulare Basin to cropland (82). A series of atmospheric rivers starting in December 1861 and continuing into 1862 generated a megaflood that inundated much of the Central Valley, flooding areas around Los Angeles and portions of the Mojave Desert (83). In response to this event, and to growing demands for water transfers and drought protection, engineering management of the two rivers began in earnest. Since 1950, nearly every major river draining the Sierra Nevada has been dammed for flood control, water supply, power, and recreation. Today California’s 1,400 large dams and tens of thousands of small dams (84) reroute flows to agricultural fields and human water supplies. These, along with diversions diking and draining in Central Valley, have eliminated >90% of California’s original four million acres of wetland (85, 86). Tulare Lake, once the largest lake west of the Mississippi, was a productive ecosystem supporting huge populations of fish, waterfowl and possibly the largest population of Native Americans north of Mexico before it was drained for agriculture (87).

In spring of 2023, however, after up to 31 atmospheric rivers triggered major flooding in California (88), about 460 km^2^ of the Tulare Lake basin refilled, destroying croplands in the southern San Joaquin Valley. Clay-rich soils under the Tulare basin will retard the infiltration of this water for at least several years. Although challenged by submerged electronic infrastructure and oil and pesticide pollution, white-faced ibis, coot, and other waterfowl are increasing in numbers around the lake. Concerningly, avian botulism (one of the plagues of waterfowl crowded into shrunken remnants of wetlands) has been detected in Tulare (89). Expanded wetlands habitat that would allow birds to socially distance should reduce such diseases (90), but if current colonists to Tulare are coming from infected populations, this benefit may be delayed.

The re-emergence of Tulare Lake within their ancestral lands is celebrated by the Tachi Yokut tribe, whose ancestors once flourished there (91). The tribe, along with environmentalists and water managers, are pointing out the advantages of sustaining the lake, not only for cultural and ecological values, but also for aquifer recharge and future floodwater dissipation (91). Under drought-deluge regimes in a future California, we should re-configure our social–ecological landscapes to realize the environmental, spiritual, and economic benefits of floodplains for humans as well as turtles, fish, tule elk, waterfowl, and other aquatic floodplain biota of California.

### Salmon Migration and Rearing in a Bypass Floodplain.

The 24,000-ha Yolo Bypass floodplain just west of the city of Sacramento demonstrates that crops and fish can successfully “timeshare” floodplains. Created to protect the city of Sacramento from flooding, the Yolo Bypass is 66 km long, 5 km wide, and at times carries 80% of the total Sacramento River discharge (92, 93). Agricultural fields make up most of the habitat in Yolo Bypass, but approximately one-third of the floodplain area is natural vegetation, including riparian and upland habitat, emergent marsh, and permanent ponds. The seasonal floodplain habitat of the Yolo diversion supports large flocks of birds (migratory geese, ducks, cranes, and resident herons, egrets, and rails), colonies of midge and mosquito-devouring bridge-roosting bats (*Tadarida brasiliensis)*, and migrating juvenile Chinook salmon (*Oncorhynchus tshawytscha*). Sommer et al. (92) compared growth of juvenile Chinook out-migrating down the mainstem Sacramento River to growth of Chinook passing through the adjacent flooded Yolo Bypass and found that floodplain fish grew significantly larger. When raised in experimental enclosures, Chinook grew five times faster on the floodplain than in the adjacent mainstem channel Sacramento River channel, as they fed on zooplankton that were >50× more abundant (94). Juvenile floodplain Chinook also gorged on copious midge larvae that emerge from inundated soils (95). For chinook and other anadromous salmonids, size at ocean entry is a key determinant of survival at sea and return as spawning adults.

Given the likelihood that climate change will increase the size and intensity, and possibly the number of atmospheric river events (96), calls to capture floodwaters to enhance water resilience in California are increasing (97). Allowing rivers to inundate their floodplains has many societal benefits appreciated since ancient times, including dissipating floodwaters, infiltration and cleansing of runoff, retaining and assimilating nutrients that might trigger harmful algal blooms downstream, restoring soil fertility, recharging groundwater, and supporting vibrant populations of wildlife, fish, waterfowl, and other native aquatic biota (98). The Yolo Bypass and other examples suggest that flexible, nimble management, with advanced planning to shift lands from farms to floodplains contingent on a year’s precipitation, could help restore tracts of California’s iconic former river and coastal floodplain ecosystems, enhancing long-term resilience of agriculture, livestock, fisheries, and water management under climate change (98).

## Natural History, Place-Based Experience, and Context-Dependency

Forecasting states of freshwater biota and ecosystems under environmental conditions beyond our present experience requires 1) long-term, place-based natural history knowledge (of biota, climate, and relevant landscapes); 2) knowledge of the interactions of biota and physical-chemical factors that can determine ecosystem states; and 3) informed hypotheses about how interactions will change under shifting environmental contexts. This knowledge, derived from scientific research, traditional ecological knowledge, or both (99), sets the stage for further tests of our understanding, including forecasting whether well-intentioned restoration actions will be helpful or harmful.

While California citizens, tribes, and agencies are ramping up efforts to enhance climate resilience in natural, restored, and human-dominated freshwater ecosystems, we stress that such efforts must rest on causal understanding—more than we generally have at present. For example, will channel modifications intended to restore floodplains instead increase sediment deposition and send flows subsurface? In 2014, the Eel River dried up near its mouth (where it drains ~9,500 km^2^) for the first time in recorded history (48). We do not yet know the relative contributions of four possible causes: warming-caused drought, summertime water extraction (100); increasing forest evapotranspiration (e.g., “Doug firification” due to fire suppression); or undergrounding of flow by excessive sediments deposited in channels after erosive land use. Where, when, and how much does each contribute to the problem? Watershed and life-history scale understanding is needed to predict which measures in which contexts (e.g., landscape position in the watershed, anticipated water and sediment supply) will be effective, and which might be ineffective or even harmful (e.g., deepening channels or adding wood structure to river channels only to have efforts buried by sediment deposition). There have been encouraging success stories. Knowledge of life history and behavioral adaptations of native western fishes to winter flood-summer drought hydrographs, and the lack of such adaptations in invasive fishes from the Mississippi basin, allowed dam managers to restore a quasi-natural flow regime to a California creek and reverse its fish faunal composition from 70% exotic-30% native to 70% native-30% exotic, with only a small, well-timed release of allocated water (101).

Finding effective restoration measures depends on modern as well as time-tested tools of geomorphology, hydrology, and field biology and ecology: long-term monitoring, surveys across environmental gradients, manipulative experiments, comparative field observations motivated by hypotheses, and quantitative or semi-quantitative models based on causal insights from all of these approaches. While quantitative process models are essential if we are to forecast ecosystem responses to progressive climate change, the shortcomings of equilibrium-based mathematical models for capturing non-linear ecological dynamics in shifting environments are well known. Alternative equation-free approaches, such as empirical dynamic modeling (102) or an older approach, frame-based modeling (103), may step us toward mechanistic prediction. The first approach fits quantitative data (e.g., population time series under environmental change) to infer interactions between environmental change and ecosystem or population responses (102). The second allows users to switch between model modules when index driver variables cross certain thresholds. These and similar approaches may accelerate forecasting useful for management and restoration in the near term and could also complement process models forecasting ecosystem responses to future change.

Finally, we note the importance of evolving water management and use strategies to protect California’s freshwater ecosystems facing changing climatic conditions and new threats. Efforts are already underway to remove some of the most ecologically damaging dams, [e.g., four dams on the Klamath River (104)], to limit minimum flows and maximum temperatures in rivers, and to re-establish wetlands for migratory birds in cooperation with farmers. Improvements in water-use efficiency have already decreased per-capita water use around the state, permitting population and economic growth while reducing total withdrawals of water from natural systems. Expansion of water treatment and recycling and stormwater capture could expand water supply without taking more water from ecosystems (105). These strategies for changing human manipulation of California’s hydrologic systems can also increase the resilience of freshwater ecosystems.

## Prediction to Guide Action: The Future of California’s Freshwater Ecosystems

As described above, many California agencies are committed to documenting, restoring, and improving the state’s freshwater ecosystems to confront climate change. Increasingly, conservationists and managers are recognizing individual species as part of an ecosystem in which interactions matter (106, 107). Correlations of species occurrence and abundance with climate-sensitive habitat attributes (e.g., flow depth, velocities, and temperature of rivers) are useful, but insufficient for prediction. Mount et al. (98) call for “ecosystem-based management” for freshwaters: “simultaneous management of water, land, and organisms to achieve a desired ecosystem condition that benefits both native biodiversity and human well-being.” This has long been practiced in California’s coastal marine environment (106, 107). In their substantial review article on climate change and ecosystems, Weiskopf et al. (108) observed that modeling remains uncertain for many ecosystems due to lack of data on “biotic interactions, community structure and function, adaptive capacity, and interactions of climate and non-climate stressors.” They call for more sophisticated models that account for “multi-species interaction, community structure, dispersal, and evolution,” even if specific predictive capability is not greatly increased. Such models may reveal causal relationships and forecast future ecosystem states not realized in simple single-species models. Ecosystem-based management of freshwaters clearly requires that we enhance our ability to predict hydrologic controls on ecosystems, especially the low summer flows that sustain California’s river ecosystems and lake levels during the seasonal peak of their biological productivity. Impacts of these hydroclimatic changes depend on what lies beneath these landscapes—the subsurface lithology, rhizosphere, and structure of the critical zone (11, 109–111) controls recharge, storage, and groundwater discharge to surface waters, so plays a crucial but poorly understood underpinning of resilience in California watersheds. A “critical zone” perspective (vertically, the zone from top of the vegetation canopy down through the soil and weathered bedrock to fresh bedrock and the depth of active groundwater, *SI Appendix*, Fig. S1 ) highlights the need to understand the dynamics of water storage and release, both in soil and in the weathered bedrock beneath (112). Just as ridgetop divides are the natural horizontal boundaries delimiting watersheds, the critical zone is their natural vertical dimension.

In response to these needs to connect ecosystem response to climate change, we suggest that numerical watershed models could explore the future trajectories of species’ populations or ecosystem functions under different climate scenarios and explore interactions with coupled ecological and social–ecological systems, whether adjacent or remote. A common model structure—connecting physical changes to ecosystem processes—could be developed, then tailored for specific locations and biota. Species interactions and food web dynamics would be important components of such models, as would hydrologic models that account for the influence of lithology on water storage and low flows in rivers in hilly and mountainous areas.

To develop this monitoring, learning, and ecosystem modeling capability, the expertise and experience of all relevant agencies, academics, tribes, and practitioners will be needed. Four components will be essential to address, anticipate, and design resilience actions before effects of future climate changes are irreversible: 1) expanding our ability to model mechanistically how freshwater biota and ecosystems respond to environmental change; 2) hypothesis-driven monitoring and field studies; 3) education and training to build research, practitioner, stewardship, and policy capabilities; and 4) developing tools and policies for building resilient ecosystems. Research described above illustrates some of these components, for example, the prediction that factors that elevate temperature in forested watersheds or deep-release outflows from dams may accelerate frog growth and development, but that these will be curtailed if warming above a certain threshold triggers virulent parasitism (51, 52). Another case of predictive ecosystem modeling comes from recent research that uncovered the seasonal migratory patterns of an invasive, warm-water piscivore, the Sacramento pikeminnow (*Ptychocheilus grandis*) (*SI Appendix*, Fig. S2 ). This discovery informed a model predicting spatio-temporal overlap of pikeminnow with young salmonids and other native prey during summers with different flow and thermal regimes (113), which in turn could inform water management during summer low-flow periods. It also motivated the Wiyot tribe, Berkeley researchers, Stillwater Sciences (private sector) and CalTrout (a non-governmental organization) restorationists to install a summertime weir across the migration pathway that has reduced pikeminnow arrivals in upstream juvenile salmonid rearing habitats (*SI Appendix*, Fig. S2 ).

Can we advance our understanding of freshwater ecosystems sufficiently so that successful a priori measures can be taken to build recovery or resilience? Climate change will continue without necessarily arriving at any new “steady state.” The goal of this four-part program is to stay ahead of changes. Building a shared numerical modeling framework will be challenging and must be sustained by decades of funding (just as climate modeling has been), but the approach could be immediately applied and iteratively tested with field work to learn and discover through adaptive management. In summary, climate change is not a step function in which we simply shift to a new condition. Changes are destined now to continue for many decades and well into the next century.

Active learning through field studies about ecosystems guided by and influencing modeling is needed for predictions to build resilience. Just the work of building a modeling framework will highlight knowledge gaps and lead to hypothesis-driven monitoring as well as expanded and focused field studies. Models can range from simple empirical approximations to process-rich numerical models. A state center focused on developing ecosystem models and their applications (i.e. “climate solutions”) could bring together tribes, agencies, academics, and other concerned citizens to share expertise and arrive at common questions and needs. Modeling and field studies go hand-in-hand, so the center could also co-ordinate long-term monitoring sites to serve as sentinel sites and test beds for hypotheses underlying proposed climate solutions hypotheses. Advanced training in ecosystem science will be needed to develop the expertise to contribute to community model development, to conduct essential remote sensing and field studies, and to work with all relevant groups to develop and implement management strategies to build resilient ecosystems. Such implementation would be done by agencies members and practitioners with strong training in ecology, freshwater and watershed ecosystem science, hydrology, and geomorphology. A science of freshwater ecosystem resilience solutions, with strong evidence of success, needs to be developed to support management applications.

Quantitative process-based modeling of freshwater ecosystems will be challenging. But just as we did not rely on conceptual models and statistics to predict climate change, we must develop mechanistic models that incorporate ecological interactions to anticipate effects of predicted climate change and to conceive of solutions that can protect and restore the freshwater ecosystems of California.

## Supplementary Material

Appendix 01 (PDF)

## Data Availability

There are no data underlying this work.

## References

[1] A. AghaKouchak, L. Cheng, O. Mazdiyasni, A. Farahmand, Global warming and changes in risk of concurrent climate extremes: Insights from the 2014 California drought. Geophys. Res. Lett. **41**, 8847–8852 (2014).

[2] S. M. Yarnell, Functional flows in modified riverscapes: Hydrographs, habitats and opportunities. BioScience **65**, 963–972 (2015).

[3] J. F. Mount, California Rivers and Streams: The Conflict Between Fluvial Process and Land Use (University of California Press, 1995).

[4] M. D. Dettinger, F. M. Ralph, T. Das, P. J. Neiman, D. R. Cayan, Atmospheric rivers, floods and the water resources of California. Water **3**, 445–478 (2011).

[5] FAQ: Climate change in California. UC San Diego, Scripps Insitution of Oceanography. https://scripps.ucsd.edu/research/climate-change-resources/faq-climate-change-california. Accessed 3 December 2023.

[6] Indicators of Climate Change in California. Precipitation (page III-47–III-60) (2022) California Office of Environmental Health Hazard Assessment. https://oehha.ca.gov/media/epic/downloads/02precip.pdf. Accessed 3 December 2023.

[7] P. H. Gleick, Regional hydrologic consequences of increases in atmospheric CO2 and other trace gases. Clim. Change **10**, 137–160 (1987).

[8] K. N. Musselman, N. Addor, J. A. Vano, N. P. Molotch, Winter melt trends portend widespread declines in snow water resources. Nat. Clim. Chang. **11**, 418–424 (2021). 10.1038/s41558-021-01014-9 PMC8098099 33968161

[9] A. T. David, J. E. Asarian, F. K. Lake, Wildfire smoke cools summer river and stream water temperatures. Water Res. Res. **54**, 7273–7290 (2018).

[10] T. J. Caldwell, S. Chandra, K. Feher, J. B. Simmons, Z. Hogan, Ecosystem response to earlier ice break-up date: Climate-driven changes to water temperature, lake-habitat-specific production, and trout habitat and resource use. Glob Change Biol. **26**, 5475–5491 (2020), 10.1111/gcb.15258. 32602183

[11] A. P. Williams, B. I. Cook, J. E. Smerdon, Rapid intensification of the emerging southwestern North American megadrought in 2020–2021. Nat. Clim. Chang. **12**, 232–234 (2022).

[12] J. Luković, J. C. Chiang, D. Blagojević, A. Sekulić, A later onset of the rainy season in California. Geophys. Res. Lett. **48**, e2020GL090350 (2021).

[13] S. J. Kelson, S. M. Carlson, Do precipitation extremes drive growth and migration timing of a Pacific salmonid fish in Mediterranean-climate streams? Ecosphere **10**, e02618 (2019).

[14] J. Howard, K. Klausmeyer, K. Fesenmyer, Below the Surface: California’s Freshwater Biodiversity (The Nature Conservancy of California, San Francisco, CA, 2013).

[15] M. S. Dogan, S. Mustafa, I. Buck, J. Medellin-Azuara, J. R. Lund, Statewide effects of ending long-term groundwater overdraft in California. J. Water Res. Plann. Manag. **145**, 04019035 (2019).

[16] T. S. Presser, The Kesterson effect. Environ. Manag. **18**, 437–454 (1994).

[17] K. R. Hultine, D. W. Bean, T. L. Dudley, C. A. Gehring, Species introductions and their cascading impacts on biotic interactions in desert riparian ecosystems. Integr. Comp. Biol. **55**, 587–601 (2015). 25908667 10.1093/icb/icv019

[18] T. Anderson, Water hyacinth thrives in drought-stricken Delta. Bay Nature (2014), https://baynature.org/article/water-hyacinth-thrives-drought-stricken-delta/. Accessed 3 December 2023.

[19] P. B. Moyle, H. W. Li, B. A. Barton, “The Frankenstein effect: Impact of introduced fishes on native fishes of North America” in The Role of Fish Culture in Fisheries Management, R. H. Stroud, Ed. (American Fisheries Society, 1987), pp. 415–426.

[20] R. M. Quiñones, P. B. Moyle, California’s freshwater fishes: Status and management. Fishes Med. Environ. 2015.001: 20p. (2015), 10.29094/FiSHMED.2015.001. Accessed 20 March 2024.

[21] P. B. Moyle, J. V. E. Katz, R. M. Quiñones, Rapid decline of California’s native inland fishes: A status assessment. Biol. Conser. **144**, 2414–2423 (2011).

[22] R. M. Yoshiyama, P. Moyle, “Historical review of Eel River anadromous salmonids, with emphasis on Chinook salmon, coho salmon and steelhead” (Report for California Trout, Center for Watershed Sciences, University of California, Davis, 2010).

[23] K. M. Norgaard, The effects of altered diet on the health of the Karuk people: A preliminary report for the Karuk Tribe of California: Department of Natural Resources Water Quality Program (Copyright: Karuk Tribe ofCalifornia, 2004).

[24] M. Roberts, A. Milman, W. Blomquist, “The sustainable groundwater management act (SGMA): California’s prescription for common challenges of groundwater governance” in Water Resilience: Management and Governance in Times of Change, J. Baird, R. Plummer, Eds. (Springer Nature Switzerland AG, 2020), pp. 41–63.

[25] California Department of Water Resources, California Water Resilience Portfolio July 2020, Governor’s Executive Order N-10-19. Public Affairs Office, Creative Services Branch (2021), p. 140.

[26] California Department of Water Resources. State Plan of Flood Control Descriptive Document (2010), p. 154.

[27] W. Arnold, A climate action plan for the California Department of Water Resources. Am. Water Works Assoc. **114**, 11–20 (2022), 10.1002/awwa.2011.

[28] Administration of Governor Gavin Newsom, Pathways to 30 x30 California, Accelerating Conservation of California’s Nature (California Natural Resources Agency, 2022), p. 72.

[29] California Department of Water Resources, Handbook for Water Budget Development, with or without models (California Department of Water Resources (DWR), 2020), p. 446.

[30] California Environmental Flows Working Group (CEFWG), California Environmental Flows Framework Version 1.0 (California Water Quality Monitoring Council Technical Report, 2021), p. 65.

[31] California Department of Water Resources, Integrated Water Flow Model, IWFM-2015.1.1443 (2023), https://water.ca.gov/Library/Modeling-and-Analysis/Modeling-Platforms/Integrated-Water-Flow-Model. Accessed 3 December 2023.

[32] California Department of Fish and Wildlife, Watershed-wide instream flow criteria for the South Fork Eel River, Instream Flow Program (CDFW), West Sacramento, CA (Watershed Criteria Report No. 2021–02, 2021). https://wildlife.ca.gov/Conservation/Watersheds/Instream-Flow/Watershed-Criteria. Accessed 3 December 2023.

[33] IPCC, “Summary for Policymakers” in Climate Change 2023: Synthesis Report. Contribution of Working Groups I, II and III to the Sixth Assessment Report of the Intergovernmental Panel on Climate Change, Core Writing Team, H. Lee, J. Romero, Eds. (IPCC, Geneva, Switzerland, 2023), pp. 1–34, 10.59327/IPCC/AR6-9789291691647.001.

[34] C. Prudhomme, N. Reynard, S. Crooks, Downscaling of global climate models for flood frequency analysis: Where are we now? Hydrol. Process. **16**, 1137–1150 (2002).

[35] A. A. Agrawal, Filling key gaps in population and community ecology. Front. Ecol. Environ. **5**, 145–152 (2007).

[36] O. Schmitz, E. Post, C. Burns, K. Johnston, Ecosystem responses to global climate change: Moving beyond color mapping. BioScience **53**, 1199–1205 (2003).

[37] K. B. Suttle, M. A. Thomsen, M. E. Power, Species interactions reverse grassland responses to changing climate. Science **315**, 640–642 (2007). 17272720 10.1126/science.1136401

[38] B. L. Peckarsky, M. E. Power “Chapter 10. The ecology of place” in Foundations of Stream Ecology, W. F. Cross, J. Benstead, A. M. Macarelli, R. Sponseller, Eds. (University of Chicago Press, Chicago, IL, 2024).

[39] Y. Vadeboncoeur, M. E. Power, Attached algae: The cryptic base of inverted trophic pyramids in freshwaters. Annu. Rev. Ecol. Evol. Syst. **48**, 255–279 (2017).

[40] M. E. Power, Effects of fish in river food webs. Science **250**, 811–814 (1990). 17759974 10.1126/science.250.4982.811

[41] J. T. Wootton, M. S. Parker, M. E. Power, Effects of disturbance on river food webs. Science **273**, 1558–1561 (1996).

[42] M. E. Power, M. S. Parker, W. E. Dietrich, Seasonal reassembly of a river food web: Floods, droughts, and impacts of fish. Ecol. Monographs **78**, 263–282 (2008).

[43] M. S. Parker, M. E. Power, “Effect of stream flow regulation and absence of scouring floods on trophic transfer of biomass to fish in Northern California rivers” (Technical Completion Report Project Number UCAL-WRC-W-825, January 1997, University of California Water Resources Center). https://escholarship.org/content/qt90f0p629/qt90f0p629_noSplash_40cb9d793bb1915c69ccd6fcf8837761.pdf?t=lnr79u. Accessed 3 December 2023.

[44] M. E. Power, Algal mats and insect emergence in rivers under Mediterranean climates: Towards photogrammetric surveillance. Freshwater Biol. **54**, 2101–2115 (2008).

[45] C. M. Ng, “The transport of chemicals and biota into coastal rivers and marine ecosystems”, PhD dissertation, University of California, Berkeley (2012), pp. 1–73.

[46] H. R. DeYoe, R. L. Lowe, J. C. Marks, Effects of nitrogen and phosphorus on the endosymbiont load of *Rhopalodia gibba* and *Epithemia turgida* (Bacillariophyceae). J. Phycol. **28**, 773–777 (1992).

[47] S. J. Kupferberg, J. Marks, M. Power, Effects of variation in natural algal and detrital diets on larval anuran (*Hyla regilla*) life-history traits. Copeia **1994**, 446–457 (1994).

[48] M. E. Power, K. Bouma-Gregson, P. Higgins, S. M. Carlson, The Thirsty Eel: Summer and winter flow thresholds that tilt the Eel River of Northwestern California from salmon-supporting to cyanobacterially degraded states. Copeia **2015**, 200–211 (2015).

[49] K. Bouma-Gregson, M. E. Power, M. Bormans, Rise and fall of toxic benthic freshwater cyanobacteria (*Anabaena* spp.) in the Eel River: Buoyancy and dispersal. Harmful Algae **66**, 79–87 (2017). 28602256 10.1016/j.hal.2017.05.007

[50] K. Bouma-Gregson, R. M. Kudela, M. E. Power, Widespread anatoxin-a detection in benthic cyanobacterial mats throughout a river network. PLoS One **13**, e0197669-21 (2018). 29775481 10.1371/journal.pone.0197669 PMC5959195

[51] S. J. Kupferberg, Effects of flow regimes altered by dams on survival, population declines, and range-wide losses of California river-breeding frogs. Conser. Biol. **26**, 513–524 (2012). 10.1111/j.1523-1739.2012.01837.x 22594596

[52] S. J. Kupferberg, A. Catenazzi, K. Lunde, A. J. Lind, W. J. Palen, Parasitic copepod (*Lernaea cyprinacea*) outbreaks in foothill yellow-legged frogs (*Rana boylii*) linked to unusually warm summers and amphibian malformations in Northern California. Copeia **2009**, 529–537 (2009).

[53] C. J. Schaaf, S. J. Kelson, S. C. Nusslé, S. M. Carlson, Black spot infection in juvenile steelhead trout increases with stream temperature in northern California. Environ. Biol. Fishes **100**, 733–744 (2017).

[54] P. Chesson, Mechanisms of maintenance of species diversity. Annu. Rev. Ecol. Syst. **31**, 343–366 (2000).

[55] R. R. Warner, P. L. Chesson, Coexistence mediated by recruitment fluctuations: A field guide to the storage effect. Amer Nat. **12**, 769–787 (1985).

[56] N. Pepin, Elevation-dependent warming in mountain regions of the world. Nat. Clim. Change **5**, 424–430 (2015).

[57] A. A. Harpold, M. D. Dettinger, S. Rajagopal, Defining snow drought and why it matters. EOS **98**, 1–6 (2017).

[58] J. Brown, Tourism is the driving force of Lake Tahoe’s $5.1 billion economy. Is it sustainable? SFGate May 11 2022, https://www.sfgate.com/renotahoe/article/How-tourism-ravages-Lake-Tahoe-17162526.php (2022).

[59] T. J. Swift, Water clarity modeling in Lake Tahoe: Linking suspended matter characteristics to Secchi depth. Aquat. Sci. **68**, 1–15 (2006), 10.1007/s00027-005-0798-x.

[60] J. J. Elser, C. R. Goldman, Zooplankton effects on phytoplankton in lakes of contrasting trophic status. Limnol. Oceanogr. **36**, 64–90 (1991).

[61] S. Chandra, Viewpoint: COMMUNICATING SCIENCE THROUGH PRESS RELEASES TO NEWS MEDIA The case study of what is controlling the fabled water clarity of Lake Tahoe. Viewpoint, Bulletin of the Amer. Soc. Limnol. Oceanogr. 1–4 (2024).

[62] A. Bess, Z. S. Chandra, S. Kelson, E. Suenega, A. Heyvaert, Zooplankton influences on the phytoplankton, water clarity, and nutrients in Lake Tahoe. Aquatic Sci. **83**, 1–15 (2021), 10.1007/s00027-020-00772-6.

[63] G. B. Sahoo, S. G. Schladow, J. E. Reuter, Effect of sediment and nutrient loading on Lake Tahoe (CA-NV) optical conditions and restoration opportunities using a newly developed lake clarity model. Water Res. Res. **46**, W10505 (2010).

[64] K. L. C. Ngai, B. J. Shuter, D. A. Jackson, S. Chandra, Projecting impacts of climate change on surface water temperatures of a large subalpine lake: Lake Tahoe, USA. Clim. Chang. **118**, 841–855 (2013).

[65] M. Winder, J. E. Reuter, S. G. Schladow, Lake warming favours small-sized planktonic diatoms. Proc. R Soc. Lond. B **276**, 427–435 (2009). 10.1098/rspb.2008.1200 PMC2581674 18812287

[66] S. W. Chisholm, “Phytoplankton size” in Primary Production and Biogeochemical Cycles in the Sea, P. G. Falkowski, E. D. Woodhead, K. Vivirito, Eds. (Plenum Press, 1992), pp. 213–237.

[67] Y. Vadeboncoeur, Blue waters, green Bottoms: Benthic filamentous algal blooms (FABs) are an emerging threat to clear lakes worldwide. BioScience **71**, 1011–1027 (2021), 10.1093/biosci/biab049. 34616235 PMC8490932

[68] K. S. Atkins, Variability in periphyton community and biomass over 37 years in Lake Tahoe (CA-NV). Hydrobiologia **848**, 1755–1772 (2021), 10.1007/s10750-021-04533-w.

[69] R. C. Naranjo, R. G. Niswonger, D. Smith, D. Rosenberry, S. Chandra, Linkages between hydrology and seasonal variations of nutrients and periphyton in a large oligotrophic subalpine lake. J. Hydrol. **568**, 877–890 (2019).

[70] M. Kamerath, S. Chandra, B. C. Allen, Distribution and impacts of warm water invasive fish in Lake Tahoe, CA-NV, USA. Aquatic Invasions **3**, 35–41 (2008).

[71] M. Wittmann, S. Chandra, K. Boyd, C. Jerde, Implementing invasive species control: A case study of multi-jurisdictional coordination at Lake Tahoe, USA. Manag. Biol. Invasions **6**, 319–328 (2015).

[72] A. Caires, S. Chandra, R. Nelson, Unique reproductive characteristics of Lake Tahoe’s *Capnia lacustra* (Plecoptera: Capniidae), a stonefly in decline. Freshwater Sci. **35**, 1291–1299 (2016).

[73] A. Caires, S. Chandra, B. Hayford, M. Wittmann, Four decades of change: Strong declines in the zoobenthos and plant community in a large lake. Freshwater Sci. **32**, 692–705 (2013).

[74] B. Hayford, A. Caires, S. Chandra, S. Girdner, Patterns in benthic diversity link lake trophic status to structure and potential function of three large, deep lakes. PLoS One **10**, e0117024 (2015), 10.1371/journal.pone.0117024. 25594516 PMC4296932

[75] S. Sadro, J. M. Melack, J. O. Sickman, K. Skeen, Climate warming response of mountain lakes affected by variations in snow. Limnol. Oceanogr. Lett. **4**, 9–17 (2019).

[76] A. P. Smits, N. W. Gomez, J. Dozier, S. Sadro, Winter climate and lake morphology control ice phenology and under-ice temperature and oxygen regimes in mountain lakes. J. Geophys. Res. Biogeosci. **126**, e2021JG006277 (2021).

[77] T. Caldwell, Drivers and projections of ice phenology in mountain lakes in the western United States derived from remote sensing. Limnol. Oceanogr. **66**, 995–1008 (2020).

[78] P. T. Strub, T. Powell, C. R. Goldman, Climatic forcing: Effects of El Nino and a small, temperate lake. Science **227**, 55–57 (1985). 17810024 10.1126/science.227.4682.55

[79] Y. Vadeboncoeur, G. Peterson, M. J. Vander Zanden, J. Kalff, Benthic algal contributions to primary production across lake size gradients: Interactions among morphometry, nutrients and light. Ecology **89**, 2452–2542 (2008). 10.1890/07-1058.1 18831175

[80] M. J. Vander Zanden, Y. Vadeboncoeur, S. Chandra, Fish reliance on littoral-benthic resources and the distribution of primary production in lakes. Ecosystems **14**, 894–903 (2011).

[81] F. Scordo, Hydroclimate variability affects habitat-specific (open water and littoral) lake metabolism. Water Res. Res. **57**, e2021WR031094 (2021), 10.1029/2021WR031094.

[82] W. G. Duffy, “Wetlands” in Ecosystems of California, H. Mooney, E. Zavaleta, Eds. (University of California Press, 2016), pp. 669–692.

[83] M. D. Dettinger, L. Ingram, Megastorms could drown massive portions of California. Sci. Am. **308**, 64–71 (2013). 23342454

[84] T. E. Grantham, J. H. Viers, P. B. Moyle, Systematic screening of dams for environmental flow assessment and implementation. BioScience **64**, 1006–1018 (2014).

[85] W. E. Frayer, D. D. Peters, H. R. Pywell, Wetlands of the California Central Valley: Status and Trends 1939 to mid0-1980’s (Portland, Oregon: U.S. Fish and Wildlife Service, 1989), p. 36.

[86] J. J. Opperman, P. B. Moyle, E. W. Larsen, J. L. Florsheim, A. D. Manfree, Floodplains Processes and Management for Ecosystems Services (University of California Press, 2017).

[87] T. F. Y. Garcia, An archae/pelago in the valley: On cultural landscapes of the southern Tulare Basin. SCA Proc. **35**, 180–186 (2022).

[88] G. Toohey, Volcano? Climate change? Bad luck? Why California was hit with 31 atmospheric river storms. Los Angeles Times, 11 April, 2023.

[89] https://apnews.com/article/avian-botulism-tulare-lake-california-birds-da71a7713bc54eada15bcc7d4c7283ca (2023). Accessed 3 December 2023.

[90] G. Gomez-Van Cortright, Birds flock to a resurrected Tulare Lake, peaking at nearly the size of Lake Tahoe. Bay Nature June 15, BayNature.org. (2023).

[91] I. James, This water needs to be protected’: California tribe calls for preservation of Tulare Lake. LA Times, 27 June 2023 (downloaded Aug. 11, 2023).

[92] T. R. Sommer, W. C. Harrell, A. M. Solger, B. Tom, W. Kimmerer, Effects of flow variation on channel and floodplain biota and habitats of the Sacramento River, California, USA. Aquat. Conserv. Mar Freshw. Ecosyst. **14**, 247–261 (2004).

[93] T. R. Sommer, M. L. Nobriga, W. C. Harrell, W. Batham, W. J. Kimmerer, Floodplain rearing of juvenile chinook salmon: Evidence of enhanced growth and survival. Canadian J. Fisheries Aquatic Sci. **58**, 325–333 (2001).

[94] C. A. Jeffres, E. J. Holmes, T. R. Sommer, J. V. E. Katz, Detrital food web contributes to aquatic ecosystem productivity and rapid salmon growth in a managed floodplain. Plos One **15**, e0216019 (2020). 32946438 10.1371/journal.pone.0216019 PMC7500630

[95] G. M. Benigno, T. R. Sommer, Just add water: Sources of chironomid drift in a large river floodplain. Hydrobiologia **600**, 297–305 (2007).

[96] V. Espinoza, D. E. Waliser, B. Guan, D. A. Lavers, F. M. Ralph, Global analysis of climate change projection effects on atmospheric rivers. Geophys. Res. Lett. **45**, 4299–4308 (2018).

[97] E. Geis, Water Always Wins (University of Chicago Press, 2022).

[98] J. Mount, A Path Forward for California’s Freshwater Ecosystems (Public Policy Institute of California Report, December, 2019). https://www.ppic.org/publication/a-path-forward-for-californias-freshwater-ecosystems/. Accessed 3 December 2023.

[99] A. J. Reid, “Two-Eyed Seeing”: An Indigenous framework to transform fisheries research and management. Fish Fish **22**, 243–261 (2021).

[100] S. Bauer, Impacts of surface water diversions for marijuana cultivation on aquatic habitat in four northwestern California watersheds. PLoS One **10**, e0120016 (2015). 25785849 10.1371/journal.pone.0120016 PMC4364984

[101] J. D. Kiernan, P. B. Moyle, P. K. Crain, Restoring native fish assemblages to a regulated California stream using the natural flow regime concept. Ecol. Appl. **22**, 1472–1482 (2012). 22908707 10.1890/11-0480.1

[102] H. Ye, Equation-free mechanistic ecosystem forecasting using empirical dynamic modeling. Proc. Natl. Acad. Sci. U.S.A. **112**, E1569–E1576 (2015). 25733874 10.1073/pnas.1417063112 PMC4386326

[103] A. M. Starfield, A. L. Bleloch, Building Models for Conservation and Wildlife Management (Macmillan, 1986).

[104] S. H. Munsch, Dam removal enables diverse juvenile life histories to emerge in threatened salmonids repopulating a heterogeneous landscape. Front. Ecol. Evol. **11**, 1188921 (2023).

[105] P. H. Gleick, H. Cooley, Freshwater scarcity. Annu. Rev. Environ. Res. **46**, 319–348 (2021).

[106] E. K. Pitkitch, Ecosystem-based fishery management. Science **305**, 346–347 (2004). 15256658 10.1126/science.1098222

[107] NOAA Fisheries, Western Regional Implementation Plan (WRIP), NOAA Fisheries Ecosystem-Based Fisheries Management Road Map (U.S. Department of Commerce, 2019), p. 25.

[108] S. R. Weiskopf, Climate change effects on biodiversity, ecosystems, ecosystem services, and natural resource management in the United States. Sci. Total Environ. **733**, 137782 (2020). 32209235 10.1016/j.scitotenv.2020.137782

[109] D. M. Rempe, W. E. Dietrich, Direct observations of rock moisture, a hidden component of the hydrologic cycle. Proc. Natl. Acad. Sci. U.S.A. **121**, 201800141 (2018). 10.1073/pnas.1800141115 PMC5856562 29490920

[110] S. M. Lovill, W. J. Hahm, W. E. Dietrich, Drainage from the Critical Zone: Lithologic controls on the persistence and spatial extent of wetted channels during the summer dry season. Water Resour. Res. **54**, 5702–5726 (2018).

[111] W. J. Hahm, Lithologically controlled subsurface Critical Zone thickness and water storage capacity determine regional plant community composition. Water Resour. Res. **55**, 3028–3055 (2019).

[112] D. N. Dralle, The salmonid and the subsurface: Hillslope storage capacity determines the quality and distribution of fish habitat. Ecosphere **14**, e4436 (2023).

[113] P. B. Georgakakos, D. N. Dralle, M. E. Power, Spring temperature predicts upstream migration timing of invasive Sacramento pikeminnow within its introduced range. Environ. Biol. Fish **106**, 2069–2082 (2023).

