# Supplementary material for: Anticipating responses to climate change and planning for resilience in California’s freshwater ecosystems
Source: Proc Natl Acad Sci U S A. 2024 Jul 29;121(32):e2310075121. doi: 10.1073/pnas.2310075121 (PMC11317582; doi:10.1073/pnas.2310075121)
Supplement: Supplementary file 1 — Appendix 01 (PDF) [file pnas.2310075121.sapp.pdf]

PNAS **Supporting Information for**

Anticipating responses to climate change and planning for resilience in California's freshwater ecosystems

Mary E. Power*[1], Sudeep Chandra[2], Peter Gleick[3], William E. Dietrich[4]

1. Department of Integrative Biology, University of California, Berkeley 94720
2. Department of Biology, University of Nevada, Reno 89557
3. Pacific Institute, 344 20th Street, Oakland, CA 946124.
4. Earth and Planetary Science, University of California, Berkeley 94720

* Corresponding author: mepower@berkeley.edu

This PDF file includes:

Figures S1, S2

Catenazzi and Kupferberg: Towards predictive mapping.

To evaluate impacts of thermal and radiation regimes on yellow legged frog tadpoles, Catenazzi and Kupferberg (SI 1) reared egg masses in identical flow-through enclosures in four streams that differed in forest canopy cover, and hence insolation: a dark, cool stream draining 2.6 km^2; a half-shaded stream draining 17 km^2; a sunny mainstem draining 130 km^2; and a very sunny stream draining 140 km^2 that ran through pasture and cultivated lands adjacent to the forested Angelo Reserve where the other three streams were located. Tadpoles hatched from eggs at the warmest 140 km^2 site grew faster and attained twice the size at full tail resorption as tadpoles growing most slowly in the coolest, smallest stream. (Interestingly, augmenting local food in the darker streams with high quality food, *Epithemia*-laden *Cladophora*, introduced from the mainstem, partially ameliorated the negative impacts of cool dark environments where the only local food was epilithic algae.) The crucial surprise, however, was that before the fastest growing, largest tadpoles from the pasture channel could emerge as froglets, they died, likely struck down by a pathogen or parasite, as dead tadpoles were also seen near these enclosures in the open river pools (SI 1), although Kupferberg had observed successful *R. boylii* metamorphosis at higher water temperatures at other river sites. These observations suggest that native frogs may tolerate or even prosper under warmer temperatures unless pathogens are present. More study of temperature and density controls of pathogens, parasites, and host interactions will be crucial for anticipating further responses of aquatic populations and ecosystems.

Figure S1

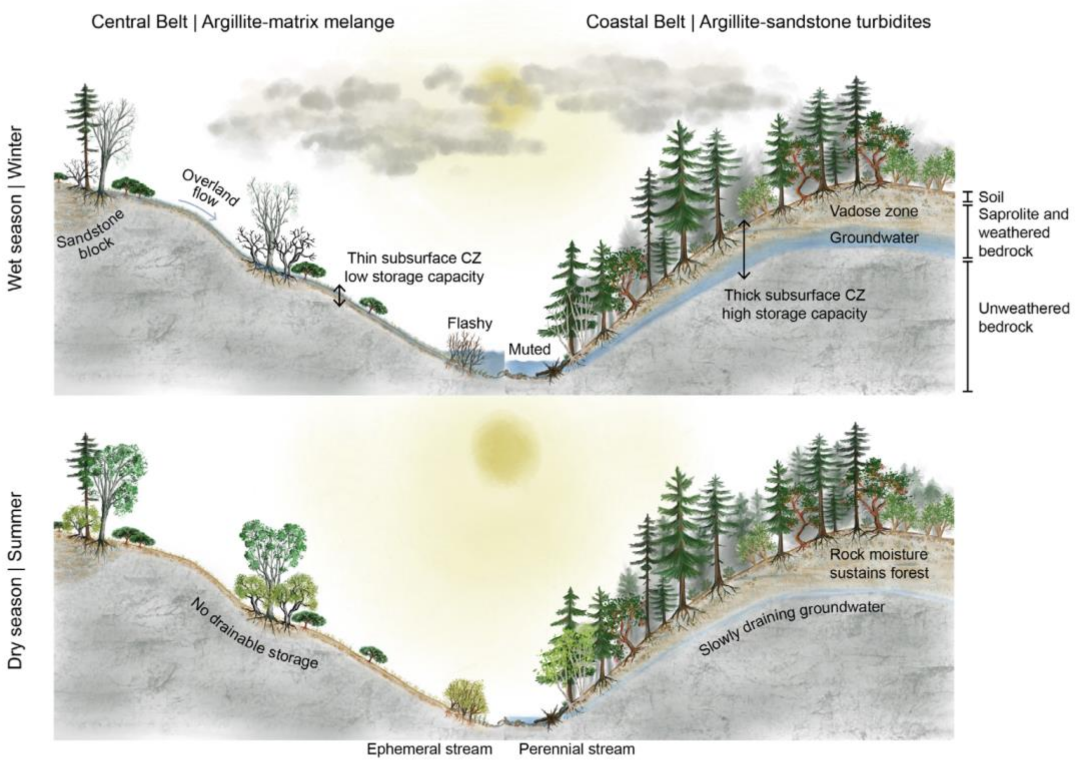

Figure S1. In hilly or mountainous landscapes, hydro-climatic controls on aquatic ecosystems are mediated in large part by the critical zone, the living skin of the Earth in which water is stored, transformed, and exchanged among vegetation, soil, and fractured weathered bedrock. Water exits the critical zone as evapo-transpiration through vegetation or as groundwater runoff that sustains surface waters after precipitation has stopped. Critical zone storage and release determines the amount, timing, and quality (temperature, chemistry) of water delivered to aquatic habitats after precipitation has ended. Lithology can control seasonal hydrologic dynamics—on the left side, clay-rich melange with shallow critical zone storage produces flashy streams that flood soon after storms and recede rapidly during dry periods. On the right side, weathered shales form deep critical zone storage that damp down flood peaks during the wet season and sustain more stable cool flows during the dry season. Image credit: Dralle et al. in https://esajournals.onlinelibrary.wiley.com/doi/full/10.1002/ecs2.4436

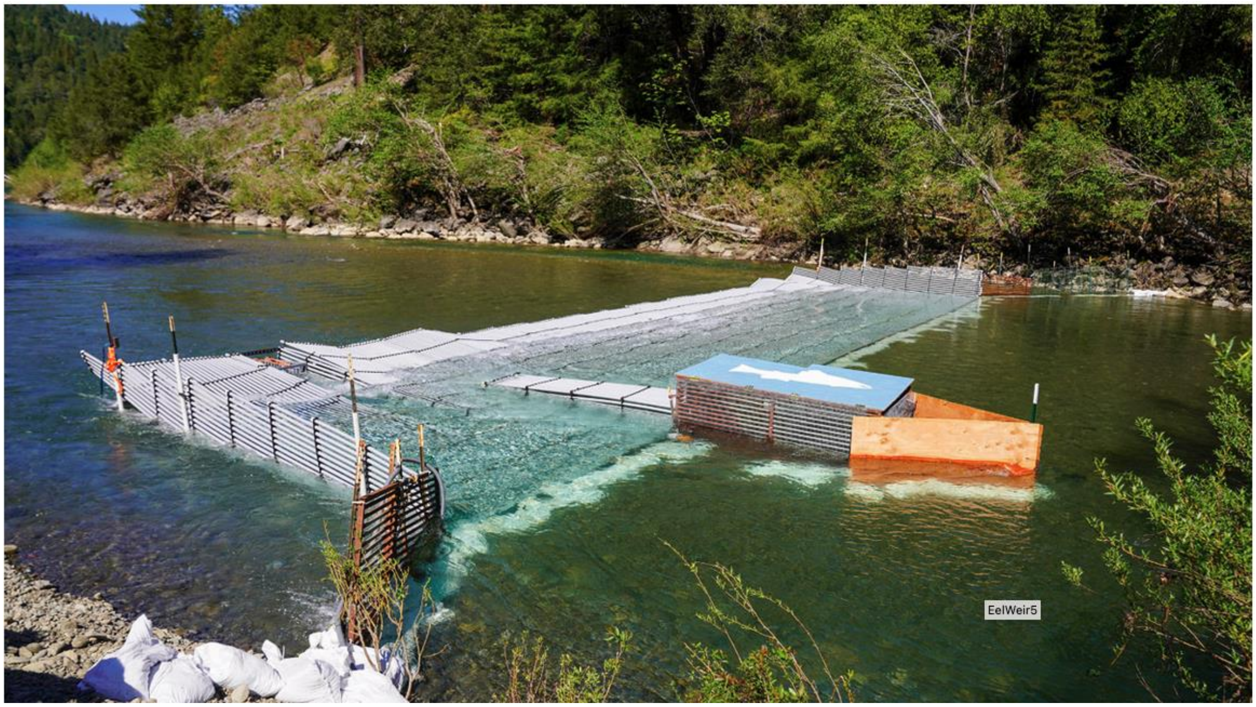

Figure S2. This channel-spanning summertime weir allows fish free passage downstream (left on photo), but traps upstream-migrating pikeminnow in a pen, where they can be captured and used in the Wiyot crab fishery or as cultural resources for other tribes. The pen is monitored continuously during the months the weir is in the river. Native vertebrates in the pen are released outside upstream of the weir after capture. The effort is a collaboration among the Wiyot, CalTrout, and U.C. Berkeley. (Photo by Gabe Rossi, used with permission. For more information, see https://caltrout.org/current-article/north-coast-science-and-monitoring-program.)

Invasive warm-water piscivores, the Sacramento pikeminnow (*Ptychocheilus grandis*), were introduced to the Eel River in 1979, spread rapidly (SI 3), and now threaten recovery of Pacific salmonids throughout the basin (SI 3-7). Georgakakos discovered that pikeminnow migrate from downriver over-wintering habitat to upstream foraging habitat in the South Fork Eel in late spring (SI 7). Repeated snorkel surveys tracking pikeminnow over up to 70 river km set the stage for Georgakakos and collaborators, including members of the Wiyot tribe whose ancestral lands encompass the lower Eel River, to trap upstream-migrating pikeminnow in a channel-spanning summertime weir, placed strategically at the time and place where and when few native vertebrates were moving. Discovering the temperature thresholds that apparently trigger pikeminnow migration allowed model forecasts of spatio-temporal overlap of these voracious piscivores with salmonids and other native prey in upstream habitats (SI 7). Spatially explicit models, tailored to specific invaded river reaches or other aquatic habitats, are increasingly feasible given advanced mapping, tracing, and sensor technology for monitoring freshwater biota (e.g. SI 8). Models forecasting potential spread and impacts of invasive species contingent on temperature or flow variables could be used, for example, to prescribe different allowable summer water extractions for warm, dry or cool, wet years (SI 7).

SI References

1. A. Catenazzi, A., S. J. Kupferberg. The importance of thermal conditions to recruitment success in stream-breeding frog populations distributed across a productivity gradient. Biological Conservation 168, 40–48 (2013).
L.R. Brown, P.B. Moyle, Invading species in the Eel River, California: successes, failures, and relationships with resident species. *Environ Biol Fish* **49**, 271–291. (1997).

2. D. N. Dralle, et al., The salmonid and the subsurface: Hillslope storage capacity determines the quality and distribution of fish habitat. Ecosphere 14 (2023).

3. L.R. Brown, P.B. Moyle, Changes in habitat and microhabitat partitioning within an assemblage of stream fishes in response to predation by Sacramento squawfish (*Ptychocheilus grandis*). *Can J Fish Aq Sci* **48**, 849–856, (1991).

4. B.C. Harvey, R. J. Nakamoto, Diel and seasonal movements by adult Sacramento pikeminnow (*Ptychocheilus grandis*) in the Eel River, northwestern California. *Ecol of Freshwater Fish* **8**, 209–215, (1999).

5. R.J. Nakamoto, B. C. Harvey, Spatial, seasonal, and size-dependent variation in the diet of Sacramento Pikeminnow in the Eel River, Northwestern California. *Ca Fish and Game* **89**, 30–45, (2003).

6. P.B. Georgakakos, Impacts of native and introduced species on native vertebrates in a salmon-bearing river under contrasting thermal and hydrologic regimes. Ph.D. dissertation, University of California, Berkeley, https://escholarship.org/uc/item/0wf2q0pk (2020).

7. PB. Georgakakos, DN. Dralle, ME. Power, Spring temperature predicts upstream migration timing of invasive Sacramento pikeminnow within its introduced range. *Environmental Biology of Fishes*, (in press).

8. G. J. Rossi, M. E. Power, S. Pneh, J. R. Neuswanger, T. J. Caldwell, Foraging modes and movements of *Oncorhynchus mykiss* as flow and invertebrate drift recede in a California stream. *Can J Fish Aquat Sci* **78**, 1045–1056 (2021).
